# Abnormal Biochemical Parameters of Macro- and Microvascular Complications in Diabetic Patients at the Bafoussam Regional Hospital of the West Region, Cameroon

**DOI:** 10.7759/cureus.91634

**Published:** 2025-09-04

**Authors:** Zadkiel Kuete Ngankeu, Guy Sedar Singor Njateng, Sammer Yousuf

**Affiliations:** 1 Biochemistry, University of Dschang, Dschang, CMR; 2 Husein Ebrahim Jamal Research Institute of Chemistry, International Center for Chemical and Biological Sciences, University of Karachi, Karachi, PAK

**Keywords:** bafoussam regional hospital, biochemical parameter, diabetes, macrovascular complication, microvascular complication

## Abstract

Background

Diabetes is a metabolic disease characterized by chronic hyperglycemia resulting from a defect in insulin secretion and/or action. It is a global health problem. When left uncontrolled over the long term, hyperglycemia can damage many body's organs, leading to disabling and potentially fatal complications such as cardiovascular disease (CVD), nerve damage (neuropathy), kidney damage (nephropathy), and eye damage, inducing retinopathy, visual loss, and even blindness. Understanding the biochemical parameters associated with diabetic complications is important for effective management and treatment. So, in order to gain insight into the incidence of these complications in diabetic patients and contribute to their better follow-up, a study was carried out at the Bafoussam Regional Hospital with the aim of assessing abnormal biochemical parameters of macro- and microvascular complications in diabetic patients.

Methodology

A descriptive cross-sectional study was conducted from April to June 2023 at the Bafoussam Regional Hospital on 113 patients of all ages. A questionnaire form was submitted to the participants, and the data from this form were analyzed. Blood samples were collected from the elbow region and used to measure biochemical parameters such as glycosylated hemoglobin (HbA1c), total cholesterol, high-density lipoprotein (HDL), low-density lipoprotein (LDL), triglycerides, creatinine, and urea.

Results

Participants were classified according to their specific characteristics and the circumstances of the discovery of diabetes. Around 70.8% diabetic patients had uncontrolled blood sugar levels revealed by a high glycated hemoglobin. HDL was high in 18.6% of patients, while it was low in 34.5%. Approximately 0.9% of patients had elevated LDL levels, while in 57.5% of patients, it was low. Triglyceride and total cholesterol levels were elevated in 37.2% and 11.5% of patients, respectively. The cardiovascular risk index and creatinine levels were elevated in 6.2% and 23% of patients, respectively. The multiple logistic regression model showed a significant association between glomerular filtration rate and neuropathy and retinopathy, as well as between creatinemia and nephropathy.

Conclusion

This study shows that poor glycemic control and longer duration of diabetes are associated with significant alterations in several biochemical parameters, which in turn are linked to an increased risk of macrovascular and microvascular complications.

## Introduction

Diabetes mellitus is defined by metabolic disturbances of various etiologies, characterized by the presence of chronic hyperglycemia, with disorders of carbohydrate, lipid, and protein metabolism, related to defects in insulin secretion, resistance to its action, or both [1]. Available statistics on diabetes in Africa testify to the scale of the challenge. Indeed, 24 million adults are currently living with diabetes, and this number is expected to increase by 129% to 55 million adults by 2045 [2]. Diabetes mellitus caused 416,000 deaths on the continent in 2021, and is expected to become one of the leading causes of death in Africa by 2030 [2]. Importantly, diabetes is the only major non-communicable disease for which the risk of premature death is increasing rather than decreasing. 

In Cameroon, specifically, the majority of health budgets are devoted to infectious diseases. However, it has to manage the burden of diabetes, which is undoubtedly the most widespread. This disease is ranked among the 15 deadliest in the world (1.5 million deaths in 2012) [3], and the fifth deadliest in Cameroon, where the prevalence is estimated to be 5.5% [4]. As a result, there is an urgent need to sensitize the entire population to the harmful consequences of this condition and how to prevent it.

The main types of diabetes are type 1, type 2, and gestational. Type 1 diabetes is caused by an autoimmune reaction in which the body's immune system attacks the insulin-producing beta cells of the pancreas. The body then produces little or no insulin, whereas type 2 diabetes is the most common type, accounting for around 90% of all diabetes cases worldwide [5]. 

If left uncontrolled over the long term, diabetes can damage many of the body's organs, leading to disabling and potentially fatal complications such as cardiovascular disease, nerve damage (neuropathy), kidney damage (nephropathy), and eye damage leading to retinopathy, visual loss, and even blindness. Nearly 90% of patients suffering from diabetes live with the disease for years without knowing, as diabetes generally causes no symptoms at the start of its course (Bencheikh et al., “Contribution to the study of some biochemical parameters in diabetics,” Master’s dissertation, Université 8 Mai 1945 Guelma, 2022). Analysis of biochemical parameters is therefore vital for screening.

A number of biochemical parameters, such as blood glucose, total cholesterol, high-density lipoprotein (HDL), low-density lipoprotein (LDL), triglycerides, creatinine, and urea, need to be analysed regularly in diabetics several times a year in order to diagnose their disease and monitor it appropriately. Strict monitoring of its evolution will help delay or prevent complications (Gamouh and Kedissa, “Comparative study of different biochemical parameters in type 1 and type 2 diabetics,” Master’s Dissertation, University of Freres Mentouri Constantine, 2016). So, this study aims at determining abnormal biochemical parameters most frequently observed in diabetic patients suffering from macro- and microvascular complications at the Bafoussam Regional Hospital. 

## Materials and methods

Design and study population 

This was a descriptive and analytical cross-sectional study conducted at the Bafoussam Regional Hospital, Bafoussam, from April to June 2023. The choice of this hospital was motivated by the fact that it is frequented by a high proportion of diabetic patients in the Western Region. The study protocol was validated by the Regional Ethics Committee for Human Health of Western Region (CRERSH-Ouest), ref: 484/30/06/2023/CE/CRERSHOU. Study participants were diabetics (type 1 and 2) of any age, regardless of sex, whose assent and consent had been obtained. 

Study site and period

This study was conducted in the Western Region of Cameroon, specifically in the Mifi Department in Bafoussam. The focal point of this study was the Bafoussam Regional Hospital, particularly the diabetology department and the clinical biology laboratories. This study was carried out over three months, from April to June 2023.

Criteria for participant selection

Inclusion Criteria

In this study, hospitalized diabetic patients or those who came for consultations to the diabetology department of the Bafoussam Regional Hospital and who provided informed consent were included. Patients for whom the pre-analytical requirements (sampling conditions) for each requested examination were met were also included.

Exclusion Criteria

Diabetic patients who refused to provide consent, as well as those with missing data, were excluded from the study.

Minimum sample size

The minimum required sample size was estimated using the Lorentz formula (N = Z2P (1-P) / M2), based on the following data: a 5.5% prevalence (P) of diabetes in Cameroon in 2022 [4], a 95% confidence interval (Z = 1.96), and a margin of error (M = 0.05), resulting in a calculated minimum sample size of 85 patients.

Blood sample collection

A questionnaire form (Appendix) was submitted to participants who had given their assent and whose consent was obtained. After disinfecting the collection site at the elbow with alcohol-soaked absorbent cotton, approximately 3 mL of blood was collected into ethylenediaminetetraacetic acid (EDTA) tubes and sodium fluoride tubes using a vacuum syringe. Blood collected in the tube containing sodium fluoride was centrifuged at 3,000 rpm for three minutes, then the supernatant was used to determine lipid profile, renal profile (urea and creatinine), and whole blood in an ethylene diamine tetraacetic acid tube to determine glycated hemoglobin (HbA1c).

Sample analysis

Biochemical examinations, including blood creatinine, blood urea, and lipid profile, were performed based on a spectrophotometric method using the semi-automated Mindray BA-88A (Mindray, Shenzhen, China). HbA1c was quantified by the HemoCue HbA1c501® hemoglobinometer (Danaher Corporation, Washington, DC).

Statistical analysis

Biochemical data and results recorded online in the data collection software kobo.humanitarianresponse.info were then transferred to Microsoft Excel (Microsoft, Redmond, WA) before being analyzed with Statistical Package for the Social Sciences version 22.0 (IBM Corp., Armonk, NY). 

Results were expressed as percentages. The P-values, odds ratio, and adjusted odds ratio of each biochemical parameter in different groups were obtained by regression testing in the model, and significant associations (P < 0.05) were detected by uni- and multivariate binary logistic regression models. The threshold values provided by the kits were used to classify the parameter as abnormal (value outside the reference range) and normal (value within the reference range). Categorical variables were described as numbers and percentages. 

## Results

This study included 113 diabetic patients of all types followed at the Bafoussam Regional Hospital. Of these patients, 42.5% were male and 57.5% were female. The mean age of the study population was 51.37 (6-85) years. The population was divided into three age groups (0-39, 40-64, and 65 and over), with the 40-64 age group the most represented, making up 45.1% of the population. There was a predominance of women (n=65; 57.5%) over men (n=48; 42.5%). Most patients had type 2 diabetes (n=85; 75.2%), and for many the disease had been discovered between the ages of 40 and 64 (n=63; 55.8%) (Table 1).

**Table 1:** Characteristics of people living with diabetes

Features	Categories		Frequency	Percentages (%)
Gender	Male		48	42.5
	Female		65	57.5
	Total		113	100
Age	0-39 years old		28	24.8
	40-64 years old		51	45.1
	65 years old and over		34	30.1
Type of diabetes	Type 1 diabetes		28	24.8
	Type 2 diabetes		85	75.2
	Total		113	100
Age of discovery	0-39 years old		40	35.4
	40-64 years old		63	55.8
	65 years old and over		10	8.8
	Total		113	100

**Figure 1:**
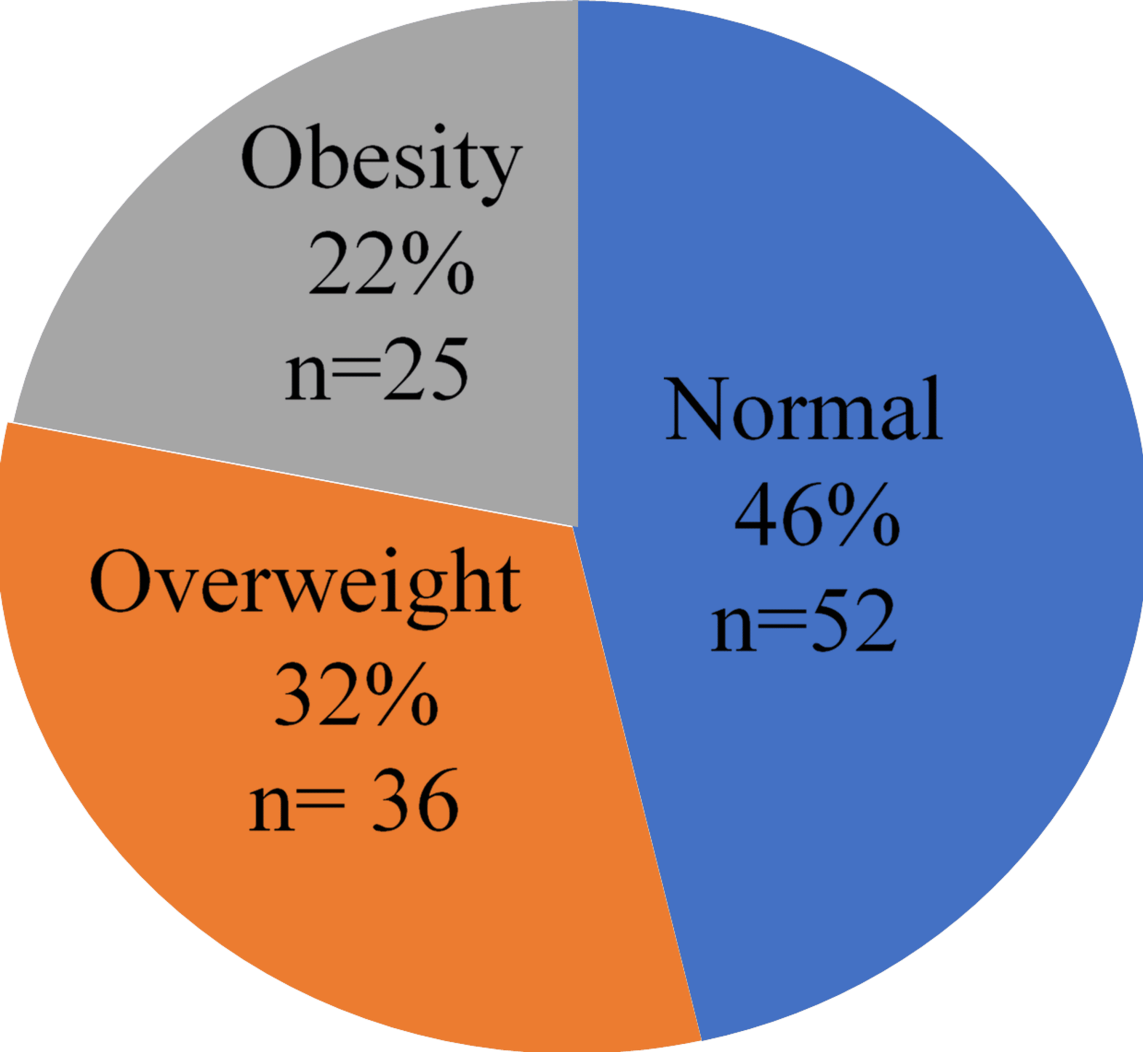
Distribution of participants according to body mass index

Figure 1 shows the distribution of participants according to their body mass index, subdivided into three classes (normal, overweight, and obese). It reveals that 46% of the population had a normal body mass index, 32% were overweight, and 22% were obese. Less than half (n=51; 46.4%) had a normal body mass index (Figure 1).

According to the circumstance of discovery of diabetes, 53.1% of cases of diabetes were discovered during consultations after experiencing symptoms (polyuria, polyphagia, polydipsia), 37.2% of cases were discovered by chance, 3.5% discovered during other circumstances not elucidated by the participants, 2.6% discovered during pregnancy and during certain complications, and finally 1% discovered during routine consultations.

Table 2 shows the proportion of diabetics with various abnormal biochemical parameters. It shows that almost three-quarters (n=80; 70.8%) of diabetics had uncontrolled blood sugar levels (high glycosylated hemoglobin). HDL and triglyceride levels were elevated in 18.6% and 37.2% diabetics, respectively. Total cholesterol levels and the risk index were elevated in 11.5% and 7.1% of participants, respectively. Around 23% (n=26) had elevated creatinine, and none had elevated urea level.

**Table 2:** Proportion of diabetics with various abnormal biochemical parameters HDL: high-density lipoprotein; LDL: low-density lipoprotein; HbA1c: glycated hemoglobin

Variables	Categories	Frequency	Percentages (%)
LDL	Low	65	57.5
	Normal	47	41.6
	High	1	0.9
	Total	113	100
HDL	Low	39	34.5
	Normal	53	46.9
	High	21	18.6
	Total	113	100
Triglycerides	Normal	71	62.8
	High	42	37.2
	Total	113	100
Total cholesterol	Normal	100	88.5
	High	13	11.5
	Total	113	100
Risk index	Low	105	92.9
	High	8	7.1
	Total	113	100
Urea	Low	98	86.7
	Normal	15	13.3
	High	0	0
	Total	113	100
Creatinine	Low	8	7.1
	Normal	79	69.9
	High	26	23
	Total	113	100
HbA1c	Normal	33	29.2
	High	80	70.8
	Total	113	100

Among diabetic patients, 40.7% had no renal failure, while 35.4% had mild renal failure (GFR=60-90 ml/min/1.73 m2); 19.5% moderate renal failure (GFR=30-59 ml/min/1.73m2); 3.5% severe renal failure (GFR=15-29 ml/min/1.73m2) and 0.9% end-stage renal failure (GFR<15 ml/min/1.73m2).

As illustrated in Table 3, in a univariate binary regression model, no abnormal biochemical parameters were associated with heart disease.

**Table 3:** Association between abnormal biochemical parameters and heart disease The chi-square test was used to compare the proportion of participants with heart disease to those without for each variable; p-value<0.005 significant. HbA1c: glycated hemoglobin; HDL: high-density lipoprotein, LDL: low-density lipoprotein, N: total number of individuals for each variable; n: number of individual per category; OR: odds ratio; GFR: glomerular filtration rate

Variables	Categories	Heart disease		P-value	Chi-square	OR (CI 95%)
		Yes, n(%); N=10	No, n(%); N=103			
HbA1c	Uncontrolled	7 (70%)	73 (70.8%)	0.855	0.003	0.89 (0.19-3.86)
	Controlled	3 (30%)	30 (29.1%)			
LDL rate	Abnormal	8 (80%)	59 (57.2%)	0.914	0.74	1.12 (0.14-9)
	Normal	3 (30%)	44 (42.7%)			
HDL rate	Abnormal	5 (50%)	34 (33.0%)	0.355	3.22	2.62 (0.36-18.75)
	Normal	5 (50%)	69 (66.9%)			
Triglyceride levels	Abnormal	5 (50%)	37 (35.9%)	0.327	0.77	2.08 (0.47-9.1)
	Normal	5 (50%)	66 (64.0%)			
Total cholesterol rate	Abnormal	1 (10%)	12 (11.6%)	0.855	0.02	1.25 (0.10-14.76)
	Normal	9 (90%)	91 (88.3%)			
Risk index	Abnormal	2 (20%)	6 (5.8%)	0.119	2.78	4.55 (0.67-30.62)
	Normal	8 (80%)	97 (94.1%)			
Urea rate	Abnormal	8 (80%)	90 (87.3%)	0.814	0.43	0.78 (0.1-6.01)
	Normal	2 (20%)	13 (12.6%)			
Creatinine	Abnormal	4 (40%)	22 (21.3%)	0.295	2.13	2.35 (0.47-11.68)
	Normal	6 (60%)	81 (78.6%)			
GFR	Abnormal	3 (30%)	60 (58.25%)	0.474	0.51	0.59 (0.14-2.44)
	Normal	7 (70%)	43 (41.74%)			

Abnormal urea (p-value=0.044) and abnormal glomerular filtration rate (p-value=0.002) were associated with neuropathy (Table 4).

**Table 4:** Association between abnormal biochemical parameters and neuropathy The chi-square test was used to compare the proportion of participants with neuropathy to those without for each variable; p-value<0.005 significant. HbA1c: glycated hemoglobin; HDL: high-density lipoprotein, LDL: low-density lipoprotein; GFR: glomerular filtration rate; N: total number of individuals for each variable; n: number of individual per category; OR: odds ratio

Variables		Categories	Neuropathy		P-value	Chi-square	OR (CI 95%)
			Yes, n(%); (N=73)	No, n(%); (N=40)			
HbA1c	Uncontrolled		56 (76.7%)	24 (60%)	0.073	3.49	2.27 (0.92-5.58)
	Controlled		17 (23.2%)	16 (40%)			
LDL rate	Abnormal		42 (57.5%)	24 (60%)	0.251	0.65	2.03(0.60-6.87)
	Normal		31 (42.4%)	16 (40%)			
HDL rate	Abnormal		21 (28.7%)	18 (45%)	0.03	3.03	0.25(0.07-0.874)
	Normal		52 (71.2%)	22 (55%)			
Triglyceride levels	Abnormal		29 (39.7%)	13 (32.5%)	0.791	0.57	0.87(0.33-2.30)
	Normal		44 (60.2%)	27 (67.5%)			
Total cholesterol rate	Abnormal		8 (10.9%)	5 (12.5%)	0.711	0.06	0.76(0.18-3.15)
	Normal		65 (89.0%)	35 (87.5%)			
Risk index	Abnormal		5 (6.84%)	3 (7.5%)	0.744	0.01	1.30(0.26-6.36)
	Normal		68 (93.1%)	37 (92.5%)			
Urea rate	Abnormal		59 (80.8%)	39 (97.5%)	0.044	6.24	0.10(0.01-0.94)
	Normal		14 (19.1%)	1 (2.5%)			
Creatinine	Abnormal		20 (27.3%)	6 (15%)	0.904	2.7	1.07(0.33-3.46)
	Normal		53 (72.6%)	34 (85%)			
GFR	Abnormal		51 (69.86%)	16 (40%)	0.002	9.54	0.28 (0.12-0.64)
	Normal		22 (30.13%)	24 (60%)			

As illustrated in Table 5, only the glomerular filtration rate was significantly associated with neuropathy (p-value=0.014 and aOR=0.35 (0.15-0.81)).

**Table 5:** Multivariate analysis of glomerular filtration rate, urea levels and neuropathy The multivariate logistics regression test was used to compare the proportion of participants with neuropathy to those without for each variable with a significant P-value in Table 4; p-value<0.005 is significant. GFR: glomerular filtration rate; N: total number of individuals for each variable; n: number of individuals per category.

Variables	Categories	Neuropathy		P-value	aOR (CI 95%)
		Yes, n(%); N=73	No, n(%); N=40		
GFR	Abnormal	51 (69.86%)	16 (40%)	0.014	0.35 (0.15-0.81)
	Normal	22 (30.14%)	24 (60%)	-	1
Urea rate	Abnormal	59 (88.82%)	39 (97.5%)	0.088	0.16 (0.02-1.31)
	Normal	14 (19.17%)	1 (2.5%)	-	1

Abnormal creatinine level (p-value=0.024) and abnormal glomerular filtration rate (p-value=0.002) were associated with retinopathy (Table 6).

**Table 6:** Association between abnormal biochemical parameters and retinopathy The chi-square test was used to compare the proportion of participants with retinopathy to those without for each variable. HbA1c: glycated hemoglobin; HDL: high-density lipoprotein, LDL: low-density lipoprotein, GFR: glomerular filtration rate; N: total number of individuals for each variable; n: number of individuals per category; OR: odds ratio; p-value<0.005 significant.

Variables	Categories	Retinopathy		P-value	Chi-square	OR (CI 95%)
		Yes, n(%); N=50	No, n(%); N=63			
HbA1c	Uncontrolled	36 (72%)	44 (69.8)	0.829	0.06	0.90(0.37-2.20)
	Controlled	14 (28%)	19 (30.1%)			
LDL rate	Abnormal	33 (66%)	33 (52.3%)	0.434	3.15	1.54(0.51-4.61)
	Normal	17 (34%)	30 (47.6%)			
HDL rate	Abnormal	19 (39%)	20 (31.7%)	0.708	0.87	1.23(0.41-3.68)
	Normal	31 (62%)	43 (68.2%)			
Triglyceride levels	Abnormal	23 (46%)	19 (30.1%)	0.090	2.99	2.16(0.88-5.29)
	Normal	27 (54%)	44 (69.8%)			
Total cholesterol rate	Abnormal	5 (10%)	8 (12.6%)	0.601	0.19	0.68(0.17-2.78)
	Normal	45 (90%)	55 (87.3%)			
Risk index	Abnormal	4 (8%)	4 (6.3%)	0.812	0.11	1.21(0.25-5.79)
	Normal	46 (92%)	59 (93.6%)			
Urea rate	Abnormal	42 (84%)	56 (88.8%)	0.702	0.57	1.29(0.34-4.89)
	Normal	8 (16%)	7 (11.1%)			
Creatinine	Abnormal	17 (34%)	9 (14.2%)	0.024	11.25	3.42(1.17-9.96)
	Normal	33 (66%)	54 (85.7%)			
GFR	Abnormal	38 (76%)	29 (46.03%)	0.002	10.37	0.26 (0.11-0.6)
	Normal	12 (24%)	34 (53.96%)			

Table 7 shows the multivariate analysis of glomerular filtration rate, creatinine level, and retinopathy. It appears that only the glomerular filtration rate was significantly associated with retinopathy (p-value=0.015).

**Table 7:** Multivariate analysis of glomerular filtration rate, creatinine level and retinopathy The multivariate logistics regression test was used to compare the proportion of participants with retinopathy to those without for each variable with a significant p-value in Table 6; p-value<0.005 significant. N: total number of individuals for each variable; n: number of individual per category; OR: odds ratio

Variables	Categories	Retinopathy		P-value	aOR (CI 95%)
		Yes, n(%); N=50	No, n(%); N=63		
Glomerular filtration rate	Abnormal	38 (76%)	29 (46.03%)	0.015	0.33(0,13-0.80)
	Normal	12 (24%)	34 (53.96%)	-	1
Creatinine rate	Abnormal	17 (34%)	9 (14.28%)	0.205	1.09 (0.70-5.15)
	Normal	33 (66%)	54 (85.71%)	-	1

Table 8 shows that no abnormal biochemical parameters were associated with stroke.

**Table 8:** Association between abnormal biochemical parameters and stroke The chi-square test was used to compare the proportion of participants with stroke to those without for each variable; p-value<0.005 significant. HbA1c: glycated hemoglobin; GFR: glomerular filtration rate; BMI: body mass index; HDL: high-density lipoprotein, LDL: low-density lipoprotein, N: total number of individuals for each variable; n: number of individual per category; OR: odds ratio

Variables	Categories	Stroke		P-value	Chi-square	OR (CI 95%)
		Yes, n(%); N=13	No, n(%); N=100			
HbA1c	Uncontrolled	11 (84.6%)	69 (69%)	0.206	1.35	2.83(0.56-14.25)
	Controlled	2 (15.3%)	31 (31%)			
LDL rate	Abnormal	7 (53.8%)	59 (59%)	0.175	0.23	0.20(0.02-2.00)
	Normal	6 (46.1%)	41 (41%)			
HDL rate	Abnormal	6 (46.1%)	33 (33%)	0.249	1.51	3.81(0.39-37.15)
	Normal	7 (53.8%)	67 (67%)			
Triglyceride levels	Abnormal	3 (23.0%)	39 (39%)	0.570	1.24	0.65(0.14-2.87)
	Normal	10 (76.9%)	61 (61%)			
Total cholesterol rate	Abnormal	0 (0%)	13 (13%)	0.999	1.91	-
	Normal	13 (100%)	87 (87%)			
Risk index	Abnormal	1 (7.69%)	7 (7%)	0.989	0.008	0.98(0.10-9.66)
	Normal	12 (92.3%)	93 (93%)			
Urea rate	Abnormal	10 (76.9%)	88 (88%)	0.611	1.22	0.63(0.10-3.69)
	Normal	3 (23.0%)	12 (12%)			
Creatinine	Abnormal	4 (30.7%)	22 (22%)	0.682	2.35	1.38(0.29-6.46)
	Normal	9 (69.2%)	78 (78%)			
GFR	Abnormal	7 (53.84%)	60 (60%)	0.672	0.18	1.28 (0.4-4.10)
	Normal	6 (46.15%)	40 (40%)			

From Table 9, it appears that triglyceride level (p-value=0.046), urea level (p-value=0.019), and creatinine (p-value=0.002) were associated with nephropathy. The other biochemical parameters were not associated with nephropathy.

**Table 9:** Association between abnormal biochemical parameters and nephropathy The chi-square test was used to compare the proportion of participants with nephropathy to those without for each variable; p-value<0.005 significant. HbA1c: glycated hemoglobin; GFR: glomerular filtration rate; BMI: body mass index; HDL: high-density lipoprotein; LDL: low-density lipoprotein; N: total number of individuals for each variable; n: number of individuals per category; OR: odds ratio; CI: confidence interval

Variables	Categories	Nephropathy		P-value	Chi-square	OR (CI 95%)
		Yes, n(%); N=67	No, n(%); N=46			
HbA1c	Uncontrolled	47(70.14%)	33(71.73%)	0.855	1.62	0.92 (0.4-2.1)
	Controlled	20 (29.85%)	13(28.26%)			
LDL rate	Abnormal	39(58.20%)	27(58.69%)	0.959	0.30	0.98 (0.45-2.09)
	Normal	28(41.79%)	19(41.30%)			
HDL rate	Abnormal	19 (28.35%)	20(43.47%)	0.099	0.21	0.51 (0.23-1.13)
	Normal	48(71.64%)	26(56.52%)			
Triglyceride levels	Abnormal	30(44.71%)	12(26.08%)	0.046	1.16	2.29 (1.01-5.19)
	Normal	37(55.22%)	34(73.91%)			
Total cholesterol rate	Abnormal	9 (13.43%)	4(8.69%)	0.441	0.499	1.62 (0.47-5.64)
	Normal	58(86.56%)	42(91.30%)			
Risk index	Abnormal	6 (8.95%)	2(4.34%)	0.395	0.19	2.16 (0.41-11.22)
	Normal	61(91.04%)	44(95.65%)			
Urea rate	Abnormal	53(79.10%)	45(97.82%)	0.019	18.60	0.084 (0.01-0.66)
	Normal	14(20.89%)	1(2.17%)			
Creatinine	Abnormal	42(62.68%)	45(97.82%)	0.002	113.00	26.78 (3.47-206.52)
	Normal	25(37.31%)	1(2.17%)			

As illustrated by Table 10, multivariate analysis of triglyceride levels, urea levels, creatinine, and nephropathy shows that only creatinine level was significantly associated with nephropathy (p-value=0.005).

**Table 10:** Multivariate analysis of triglyceride levels, urea levels, creatinine and nephropathy The multivariate logistics regression test was used to compare the proportion of participants with nephropathy to those without for each variable with significant p-values in Table 9; p-value<0.005 is significant. N: total number of individuals for each variable; n: number of individuals per category; OR: odds ratio; CI: confidence interval

Variables	Categories	Nephropathy		p-value	aOR (CI 95%)
		Yes, n(%); n=67	No, n(%); n=46		
Triglyceride levels	Abnormal	30(44.77%)	12(26.08%)	0.086	2.09(0.9-5.19)
	Normal	37(55.22%)	34(73.91%)	-	1
Urea rate	Abnormal	53(79.10%)	45(97.82%)	0.205	0.09(0.01-0.73)
	Normal	14(20.89%)	1(2.17%)	-	1
Creatinine	Abnormal	42(62.68%)	45(97.82%)	0.005	19.17(2.40-152.66)
	Normal	25(37.31%)	1(2.17%)		

Table 11 shows that the majority of participants with heart disease (n=10) were female, with a frequency of 70% (n=7), and the age group most affected by this disease was 65 and over, with a frequency of 40% (n=4) of cases. Participants with a duration of diabetes of more than 10 years were the most affected, with a frequency of 50% (n=5). The 40% (n=4) normal body mass index group had a higher incidence of heart disease than other body mass index groups. Finally, the type of diabetes with the highest incidence was type 2 diabetes, with 60% (n=6) of cases of heart disease. The majority of participants with diabetic foot (n=3) were female, with a frequency of 66.6% (n=2), and the age groups most affected by this disease were 65 and over, with a frequency of 66.6% (n=3) of cases. Participants with a duration of diabetes of more than 10 years were the most affected, with a frequency of 66.6% (n=2). The obese body mass index bracket had a higher frequency of 66.6% (n=2). Finally, the type of diabetes with the highest frequency was type 2 diabetes, with 100% (n=3) cases of diabetic foot. The majority of participants with at least one stroke (n=13) were female, with a frequency of 53.8% (n=7), and the age group most affected by stroke was the 40-64 age group, with a frequency of 53.8% (n=7) (Table 11). Participants with a duration of diabetes of 5-10 years and over 10 years were the most affected, with the same frequency of 38.44% (n=5). In this study, it was the normal body mass index group that had the highest stroke frequency, at 38.4% (n=5). Finally, the type of diabetes with the highest frequency was type 2 diabetes, with 84.6% (n=11) cases of stroke (Table 11).

**Table 11:** Distribution of macrovascular complications N: total number of individuals for each variable; n: number of individual per category

Variables	Categories	Heart disease, n(%); n=10	Diabetic feet, n(%); n=3	Stroke, n(%); n=13
Gender	Female	7 (70%)	2 (66.6%)	7 (53.8%)
	Male	3 (30%)	1 (33.3%)	6 (46.1%)
Age	Under 40	3 (30%)	0 (0%)	1 (7.6%)
	40-64 years	3 (30%)	1 (33.3%)	7 (53.8%)
	65 and over	4 (40%)	2 (66.6%)	5 (38.4%)
Duration of diabetes	Less than 5 years	3 (30%)	1 (33.3%)	3 (23.0%)
	5-10 years	2 (20%)	0 (0%)	5 (38.4%)
	Over 10 years	5 (50%)	2 (66.6%)	5 (38.4%)
Body mass index	Normal	4 (40%)	1 (33.3%)	5 (38.4%)
	Overweight	3 (30%)	0 (0%)	3 (23.0%)
	Obesity	3 (30%)	2 (66.6%)	5 (38.4%)
Type of diabetes	DT1	4 (40%)	0 (0%)	2 (15.3%)
	DT2	6(60%)	3 (100%)	11 (84.6%)

Table 12 shows the distribution of microvascular complications according to sex, age, duration of diabetes, body mass index, and type of diabetes. In this study population, 67 people suffered from nephropathy (kidney failure), with women accounting for a high frequency of 61.2% (n=41). The age group most affected was 40-64, with a frequency of 34 (50.74%). The population with a duration of diabetes of more than 10 years was more affected, with a frequency of 29 (43.3%). The body mass index with the highest frequency of nephropathy (n=25; 37.3%) was normal, and finally, the type of diabetes with the highest frequency of this disease was type 2 diabetes, with n=62 (92.5%). In the study population, 50 people suffered from retinopathy, and among them, the sex with the highest frequency was female, 64% (n=32). The age group most affected was the 40-64 age group, 54% (n=27); and the population with diabetes duration ranging from 5-10 years was more affected, 36% (n=18). The body mass index with the highest frequency of retinopathy (42% (n=21)) was normal, and finally, the type of diabetes with the highest frequency of this disease was type 2 diabetes, 90% (n=45). Moreover, 73 people suffered from neuropathy (diabetic complication with the highest frequency), and among them, the sex with the highest frequency was female, 60.2% (n=44). The age group most affected was the 40-64 age group with 43.8% (n=32); and the population with a diabetes duration of more than 10 years was more affected (35.6%; n=26). The body mass index with a high frequency of retinopathy (47.9%(n=35)) was normal, and finally, the type of diabetes with the highest frequency of this disease was type 2 diabetes, 79.4% (n=58).

**Table 12:** Distribution of microvascular complications T1D: type 1 diabetes; T2D: type 2 diabetes; N: total number of individuals for each variable; n: number of individual per category

Variables	Categories	Nephropathy, n(%); n=67	Retinopathy, n(%); n=50	Neuropathy, n(%); n=73
Gender	Female	41 (61.2%)	32 (64%)	44 (60.2%)
	Male	26 (38.8%)	18 (36%)	29 (39.7%)
Age	Under 40	4 (5.9%)	2 (4%)	15 (20.5%)
	40-64 years	34 (50.74%)	27 (54%)	32 (43.8%)
	65 and over	29(43.2%)	21 (42%)	26 (35.6%)
Duration of diabetes	Less than 5 years	16(23.9%)	11 (22%)	22 (30.1%)
	5-10 years	22(32.8%)	18 (36%)	25 (34.2%)
	Over 10 years	29(43.3%)	21 (42%)	26 (35.6%)
Body mass index	Normal	25(37.3%)	21 (42%)	35 (47.9%)
	Overweight	24(35.8%)	19 (38%)	24 (32.9%)
	Obesity	18 (26.8%)	10 (20%)	14 (19.2%)
Type of diabetes	T1D	5 (7.5%)	5 (10%)	15 (20.5%)
	T2D	62 (92.5%)	45 (90%)	58 (79.4%)

**Figure 2:**
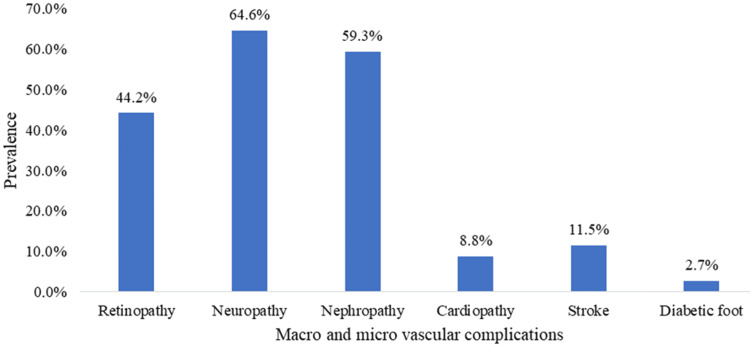
Prevalence of macro- and microvascular complications

Figure 2 shows that 98.2% (n=111) of participants had at least one macro- or microvascular complication. Macrovascular complications were less prevalent, with 11.5% (n=13) of participants having had at least one stroke; 8.8% (n=10) participants having heart disease, and 2.7% (n=3) having a diabetic foot. Microvascular complications were more prevalent, with 64.6% (n=73) suffering from neuropathy, 44.2% (n=50) from retinopathy, and 59.3% (n=67) from nephropathy. 

## Discussion

The aim of this study was to evaluate abnormal biochemical parameters in diabetic patients with macro- and microvascular complications at the Bafoussam Regional Hospital. In the course of this study, HbA1c, the main parameter for monitoring and controlling glycemia in diabetic patients, blood urea and creatinine to assess renal complications, and finally the lipid profile to assess cardiovascular complications were assayed.

Most of the patients in this study suffered from type 2 diabetes. These results are confirmed by the findings of Bencheikh et al. (2022) and Gamouh and Kedissa (2016), who found that the number of type 2 diabetics is higher, with a percentage of 65%. According to the International Diabetes Federation (IDF) Atlas, the number of people with type 2 diabetes worldwide is rising rapidly, and this increase is linked to economic development, an aging population, and growing urbanization [4]. 

In this study, the mean age was 51.37 years. This result is similar to those found by other authors in Africa, such as Mossi et al. [6] in Lome, Togo. On the other hand, in France, the average age of diabetic patients was higher (64 years) according to the study performed by Detournay et al. [7]. This confirms the young age of African diabetics, which may be due to rapid lifestyle changes in Africa, such as urbanization, access to processed foods, and the adoption of sedentary behaviors [8].

The predominance of women over men found in our study is a phenomenon confirmed by several authors, such as Mbaye et al. [9] and Sow et al. [10] in Senegal. The lack of physical activity among women, the change in traditional lifestyles, and increasing urbanization may explain this difference, as well as the fact that women are more likely than men to consult their doctor on a weekly basis, and that they are more prone than men to anxiety and stress, as elucidated by Badache et al. ("Epidemiological approach to diabetes: Interrelation of stress, diet, and hypertension in the Jijel region," Master’s dissertation, University of Jijel-Algeria, 2019). In addition, female physiology is different from that of men due to the difference in the type and rate of hormone production (Badache et al., 2019).

Around 53.1% of the participants' diabetes cases were discovered during consultations after experiencing symptoms (polyuria, polyphagia, polydipsia), and 37.2% of cases were discovered by chance. This is confirmed by the IDF [11] report, which showed that over two-thirds (69.2%) of adults with diabetes in sub-Saharan Africa remain undiagnosed. The reasons cited are limited resources and the low priority given to diabetes screening [11].

According to the results obtained, a predominance of subjects belonging to the 40-64 age group was noted. The prevalence of diabetes in this study is comparable to the results of the study conducted by Abdelkebir and Attalah ("Biological markers of complications of diabetes mellitus," Master's dissertation, Université Frères Mentouri, Constantine, Algeria, 2017). On the other hand, these results are at odds with those of the study conducted by Ouadjed (“Epidemiological study on the effect of type 2 diabetes in the progression of Alzheimer’s disease,” Master’s dissertation, Université Abdelhamid Ben Badis - Mostaganem, 2017), which showed a predominance of the 60-70 age group (36.73%) and those of the study conducted by Bencheikh et al. (2022), which showed a predominance of the 60-75 age group (32.20%). This difference can be explained by the fact that aging is accompanied by changes in carbohydrate metabolism, favoring the onset of diabetes: reduced insulin secretion. Indeed, the β-cells of the pancreas respond less well to carbohydrate stimuli, and by the reduced secretion of hormones against regulation, notably glucagon [12]. Decreased insulin sensitivity is partly explained by changes in body composition, with a decrease in lean body mass and an increase in visceral fat [13].

The distribution of participants according to their body mass index, subdivided into three classes (normal, overweight, and obese), showed that 22% of the population were obese. The frequency of obesity seems to vary according to African region: 22.5% in Senegal [10], 20% in Côte d'Ivoire [14], and 35% in Togo [6]. This variability may be due to environmental factors, dietary habits, or the different gene pools of each population.

Data analysis revealed a prevalence of 98.2% of macro- and microvascular complications of diabetes. This is higher than that obtained by Mossi et al. [6] in Lomé, Togo (26.76%). This high frequency could be due to the poor glycemic control observed in the patients and poor screening. Indeed, HbA1c in our series was above normal in 70.8% of cases. According to several epidemiological studies, microvascular complications are associated with more severe glycemic imbalance. This was highlighted, in particular, in the UKPDS (UK prospective diabetes study), which revealed that the mean HbA1c level during an 11-year follow-up was linked to a higher incidence of retinopathy, nephropathy, and neuropathy [3]. Formal evidence for the efficacy of glycemic control in preventing the microvascular complications of diabetes comes from intervention studies. Intensive insulin treatment of type 1 diabetics reduced the occurrence of retinopathy, nephropathy, and neuropathy at five years by 75%, 40%, and 70%, respectively, compared with conventional treatment, and the progression of these complications by more than 50% at five years [15].

Microvascular complications were more prevalent, with 64.6% of people suffering from neuropathy, 44.2% from retinopathy, and 23% from nephropathy. The prevalence of diabetic neuropathy varies from 0-93% depending on the study [16]. There are several reasons for this disparity: clinical symptoms are not specific to diabetic neuropathy, prevalence depends on the diagnostic criteria used and whether or not electrophysiological tests of varying sensitivity are used, nerve conduction velocities decrease physiologically with age, and nerve fibers of different types may be affected [16]. Pirart's study [17], although older, is still a reference. Involving some 4,500 patients, neuropathy is defined as the absence of Achilles reflexes associated with symptoms or objective signs of polyneuritis. The prevalence of neuropathy increases with the duration of diabetes: 7% when the onset of diabetes was less than one year ago, 50% after 20 years. These findings are corroborated by Young et al. [18]. Regardless of glycemic control, around 50% of patients do not develop clinical neuropathy, even after 20 years. On the other hand, patients with good metabolic control may develop disabling neuropathy early after the diagnosis of diabetes. This suggests the existence of factors independent of the hyperglycemic state in the pathophysiology of neuropathy. The prevalence of neuropathy is high in certain populations (Indians, North Africans). The high prevalence of retinopathy in our study is contrary to that obtained by Sobngwi et al. [19] in Cameroon. This variability in prevalence may be due to the fact that the prevalence of retinopathy in diabetic populations on the African continent varies from 16 to 55%, depending on the clinical site, duration, and control of diabetes in the study population [20]. The prevalence of nephropathy in this population was 59.3%, which is higher than that obtained by Kadiki et al. [21] (25.2%) in 1999 in Libya. This high prevalence may be due to the lack of glycemic control in this study population [21].

Macrovascular complications were present with 11.5% of participants having had at least one stroke, 8.8% participants with heart disease, and 2.7% with a diabetic foot. These results are close to those of the study of Einarson et al. [22]. This may be explained by the fact that in diabetes, excess glucose in the blood can bind to proteins, a process known as glycation. Protein glycation can lead to the formation of advanced glycation end products (AGEs), which are toxic to the endothelial cells of blood vessels [23]. This can lead to endothelial dysfunction, inflammation, and increased vascular permeability, favoring the development of macrovascular complications [24]. Diabetes can also increase the production of free radicals in the body, leading to increased oxidative stress. Oxidative stress damages cells and tissues, including blood vessel walls [25]. This can contribute to inflammation, the formation of atherosclerotic plaques, and the progression of macrovascular complications [24].

Nearly three-quarters of participants in this study had uncontrolled blood glucose levels (high HbA1c). These unfavorable results are similar to those obtained in studies by Mossi et al. [6] in Lomé (87.65%). The impact of glycemic imbalance could explain certain micro-angiopathic, macro-angiopathic, and mixed complications of diabetes. This may be due to participants' lack of information, the low level of education in this population, or even socio-demographic characteristics such as precarious socio-economic level, low level of education, access to healthcare, lack of family and social support, cultural and religious factors, and finally age and gender.

The results of the analysis of lipid profile parameters in the diabetic subjects indicate high total cholesterol and triglyceride values, while abnormally, HDL values are low. These results are similar to those of Bencheikh et al. (2022) and those of Ram et al. [14], who found an increase in cholesterol and triglyceride levels in diabetic subjects and a decrease in HDL. The main cause of these lipid changes in diabetics is insulin resistance, i.e., when the body does not respond correctly to insulin or does not produce enough of it. Insulin is a hormone that regulates glucose and lipid metabolism in the body [26]. Insulin resistance leads to chronic hyperglycemia (high blood sugar levels), which in turn can increase triglyceride levels. Excess glucose is converted into triglycerides and stored in fat cells, leading to increased blood levels. In addition, insulin resistance disrupts cholesterol metabolism, which can lead to an increase in total cholesterol [27]. 

As far as lower high-density lipoprotein levels are concerned, this can be caused by several factors associated with diabetes. One of these is hyperinsulinemia, which occurs when the body produces too much insulin to compensate for insulin resistance. This can adversely affect high-density lipoprotein levels [28]. In addition, diabetes can also impair the functioning of enzymes involved in high-density lipoprotein metabolism, reducing its production and levels in the blood [29]. It's important to note that obesity, an unbalanced diet, lack of physical activity, and a family history of dyslipidemia can also contribute to these lipid problems in diabetics [30]. The reduced LDL levels may be due to the fact that some of the participants may have been on medication, as some drugs used to treat diabetes or cholesterol problems, such as statins, can help reduce LDL levels [31].

Elevated blood creatinine values were noted in 23% of participants. These results corroborate those of Bencheikh et al. (2022), who found that mean creatinine values are above 12 mg/l in diabetics. Diabetes can damage the blood vessels of the kidneys, leading to renal dysfunction [32]. Increased creatinine levels indicate reduced glomerular filtration rate, and therefore renal failure [33].

None of the participants had high uraemia (13.2% normal and 86.7%). These results are in agreement with the findings of Bencheikh et al. (2022), who found that the majority of diabetic patients have normal blood urea levels. These results could be explained by the fact that, in patients with nephropathy, the kidneys allow certain molecules to pass through in excess, such as proteins, whose degradation leads to the production of urea in the blood. This may also be due to hypoproteinemia, caused by the loss of proteins in the urine, or to dehydration. 

In contrast to expectations, some risk factors identified in the review did not show causal links with the abnormal biochemical parameters of macro- and microvascular complications observed in our diabetic patients. But at least in one model, the duration of diabetes was associated with abnormal urea levels (p-value=0.003).

Elevated urea levels and decreased glomerular filtration rate were associated with neuropathy, and in multivariate analysis, only glomerular filtration rate was significantly associated with neuropathy. Although the relationship is not well understood, it is important to note that diabetic neuropathy is a multifactorial complication that can be influenced by many other factors, such as glycemic control, duration of diabetes, hypertension, and other cardiovascular risk factors [18].

 Abnormal creatinine level and abnormal glomerular filtration rate were associated with retinopathy, and in multivariate analysis, only glomerular filtration rate was significantly associated with retinopathy. Possible biological mechanisms that could explain this association are the fact that renal failure due to diabetic nephropathy can lead to creatinine accumulation in the blood, which can result in elevated blood creatinine levels. This, in turn, may be associated with retinopathy due to the systemic impact of diabetes on the blood vessels. Measurement of glomerular filtration rate and creatinine levels can be used as predictive indicators of the presence or progression of diabetic retinopathy [19].

These results within the univariate binary logistic regression framework showed an association between triglyceride level, urea level, and creatinine levels. Multivariate analysis was used to adjust for potential confounders; the results showed that only creatinine was significantly associated with nephropathy. This association may be explained by the fact that elevated triglycerides contribute to lipid accumulation in renal tissues, resulting in inflammation and impaired renal function, leading to reduced glomerular filtration rate and consequent retention of blood urea and creatinine by the kidney, which contributes to their increased renal levels (Abdelkebir and Attalah, 2017).

Limitations of the study

An increase in the study period, leading to a large population size and the dosage of other biochemical parameters, can allow us to observe all the abnormal biochemical parameters linked to micro and macrovascular complications in diabetic patients.

## Conclusions

This study shows that macro- and microvascular complications through abnormal biochemical parameters are related to poor glycemic control, body mass index, type of diabetes, and duration of diabetes onset. These results underline the importance of optimal glycemic control and management of lipid risk factors in diabetic patients to prevent or delay the development of these complications. In addition, regular monitoring of renal function is necessary for early detection and treatment of diabetic nephropathy.

## Appendices

The questionnaire provided to the participants is provided in Table 13.

**Table 13:** Questionnaire form

No.	Questions	Propositions	Answer
Section 1: Socio-anthropometric characteristics			
1.1	Gender	1-M; 2-F	
1.2	Age (Years)	_____________	
1.3	Weight (Kgs)	_____________	
Section 2. Detecting neuropathic pain: To estimate the probability of neuropathic pain, the patient must answer each item in the 4 questions below with “yes” or “no”.			
2.1	Does the pain have one or more of the following characteristics?	Burn	
		Painful cold sensation	
		Electric shock	
2.2	Does the pain have one or more of the following characteristics?	Stinging	
		Tingling	
		Numness	
		Itching	
2.3	Is the pain localized in a territory where the examination reveals :	Hypoesthesia to touch	
		Hypoesthesia to pricking	
2.4	Is the pain provoked or increased by	Friction	
Section 3: History of the disease			
3.1	At approximately what age did a doctor first tell you that you had diabetes (or in what year)?	_____________	
3.2	What type of diabetes do you suffer from?	1-Type 1; 2-Type 2; 3-other; 4- I don’t know	
3.3	Under what circumstances was your diabetes discovered? Tick the correct answer (several answers possible)	Because you were always thirsty and/or had to urinate and/or lost weight	
		Because you had suffered a diabetic coma	
		Because you had a problem with your heart, arteries, kidneys, nerves, eyes or feet	
		When monitoring excess weight	
		By chance, during an occupational health, social security or pre-operation check-up	
		During or after pregnancy	
		On a blood or urine test carried out for another reason	
		Other circumstances, please specify	________
Section 4: Micro and macro vascular complications			
4.1	Heart And Blood Vessels. Tick the correct answer (several answers possible)	Has a doctor ever told you that you have hypertension, or high blood pressure?	No/Yes/I don't know
		Has a doctor ever told you that you have high cholesterol or triglycerides(fats) in your blood?	No/Yes/I don't know
		Has a doctor ever told you that you have heart failure?	No/Yes/I don't know
		Has a doctor ever told you that you had a myocardial infarction, a heart attack, angina, or chest pain (coronary problem)?	No/Yes/I don't know
		Have you ever had an intervention on your heart arteries (coronary bypass surgery, coronary angioplasty, stent placement, or coronary dilation)?	No/Yes/I don't know
		Has a doctor ever told you that you had a stroke (CVA)?	No/Yes/I don't know
4.2	Eyes. Tick the correct answer (several answers possible)		
	Is your ophthalmologist aware of your diabetes?	No; if no, what are the reasons? (multiple answers possible)	He/She did not ask me; I did not want to tell him/her; Other reason, please specify:
		Yes	
		I don't know	
	Has an eye specialist ever performed a fundus examination (that is, an exam that requires putting drops in your eyes to dilate your pupils) or taken a photograph of your retina using a special device called a "retinograph"?	No	
		Yes, if yes, was it? (Multiple answers possible): 1A fundus examination (with drops). Was it?	In the last 12 months; more than 12 months ago
		I don't know	
	Have you ever received laser treatment for your eyes?	No	
		Yes, if yes, was it? (Multiple answers possible)	For your diabetes; For another issue, please specify: .........................
		I don't know	
	Has a doctor ever told you that your diabetes has affected your eyes or that you have retinopathy?	No	
		Yes	
		I don't know	
	Do you have vision problems (related to your diabetes or not)?	Reading	
		Taking your medication or giving your injections	
		Other, please specify :	…………………..
		No difficulties	
	Have you permanently lost vision in one eye?	No	
		Yes, if yes, was it (multiple answers possible)?	…………………….
		I don't know	
